# Average and Interindividual Effects to a Comprehensive Cardiovascular Rehabilitation Program

**DOI:** 10.3390/ijerph20010261

**Published:** 2022-12-24

**Authors:** Marcelo Tuesta, Cristian Alvarez, Oneglio Pedemonte, Oscar F. Araneda, Pablo Manríquez-Villarroel, Paulina Berthelon, Alvaro Reyes

**Affiliations:** 1 Exercise and Rehabilitation Sciences Institute, School of Physical Therapy, Faculty of Rehabilitation Sciences, Universidad Andres Bello, Santiago 7591538, Chile; 2 Laboratory of Cardiorespiratory Physiology, Center of Cardiovascular Rehabilitation, Dr. Jorge Kaplan Meyer Foundation, Viña del Mar 2520605, Chile; 3 Cardiovascular Surgery Department, Hospital Dr. Gustavo Fricke, Viña del Mar 2570017, Chile; 4 Laboratory of Integrative Physiology of Biomechanics and Physiology of Effort (LIBFE), Faculty of Medicine, Kinesiology School, Universidad de los Andes, Santiago 7620086, Chile; 5 School of Nutrition, Faculty of Health Sciences, Universidad Viña del Mar, Viña del Mar 2572007, Chile

**Keywords:** heart diseases, physical exercise, cardiovascular rehabilitation, concurrent exercise

## Abstract

Background: To describe the average effects and the interindividual variability after a comprehensive outpatient cardiovascular rehabilitation (CCR) program using concurrent exercise training prescribed according to cardiovascular risk stratification on cardiorespiratory fitness (CRF), anthropometric/body composition, quality of life and emotional health in patients of four cardiovascular disease profiles. Methods: CRF, anthropometric/body composition, quality of life, and emotional health were measured before and after a CCR and analyzed in heart valve surgery (HVS), heart failure with reduced ejection fraction (HFrEF), post-acute myocardial infarction (post-AMI), and in coronary artery disease (CAD) patients. Twenty, twenty-four, and thirty-two exercise sessions were prescribed according to mild, moderate, and severe baseline cardiovascular risk, respectively. In addition to concurrent exercise training, nutritional counseling, psychological support, and lifestyle education programs were performed. Results: The main outcomes by delta changes comparisons (Δ) revealed no significant differences at anthropometric/body composition as ΔBody fat decreases (HVS Δ−1.1, HFrEF Δ−1.0, post-AMI Δ−1.4, CAD Δ−1.2 kg) and ΔSkeletal muscle mass increases (HVS Δ+1.4, HFrEF Δ+0.8, post-AMI Δ+0.9, CAD Δ+0.9 kg), and CRF performance as ΔVO2peak increases (HVS Δ+4.3, HFrEF Δ+4.8, post-AMI Δ+4.1, CAD Δ+5.1 mL/kg/min) outcomes among HVS, HFrEF, post-AMI, and CAD (*p* > 0.05). Secondary outcomes showed significant pre-post delta changes in METs (HVS Δ+1.8, HFrEF Δ+0.7, post-AMI Δ+1.4, CAD Δ+1.4), and maximal O_2_pulse (HVS Δ+3.1, post-AMI Δ+2.1, CAD Δ+1.9). In addition, quality of life had a significant improvement in physical functioning (HVS Δ+17.0, HFrEF Δ+12.1, post-AMI Δ+9.8, CAD Δ+11.2), physical role (HVS Δ+28.4, HFrEF Δ+26.8, post-AMI Δ+25.6, CAD Δ+25.3), vitality (HVS Δ+18.4, HFrEF Δ+14.3, post-AMI Δ+14.2, CAD Δ+10.6) and social functioning (HVS Δ+20.4, HFrEF Δ+25.3, post-AMI Δ+20.4, CAD Δ+14.8) in all cardiovascular disease. For anxiety (HVS Δ−3.6, HFrEF Δ−2.3, post-AMI Δ−3.0, CAD Δ−3.1) and depression (HVS Δ−2.8, HFrEF Δ−3.4, post-AMI Δ−3.2, CAD Δ−2.3) significant changes were also observed. Conclusions: A CCR program that prescribes the number of exercise sessions using a cardiovascular risk stratification improves CRF, QoL, and emotional health, and the average results show a wide interindividual variability (~25% of non-responders) in this sample of four CVD profile of patients.

## 1. Introduction

Comprehensive outpatient cardiovascular rehabilitation (CCR) is essential in the management of patients with cardiovascular disease (CVD). Evidence from systematic reviews and randomized controlled trials reveal that CCR is beneficial to decreasing mortality and hospital re-admission, and improving quality of life (QoL) in individuals with different CVD [1,2]. Improvement in cardiorespiratory fitness (CRF) is pivotal to achieving these benefits, and several settings of exercise training had a well-reported role in improving this outcome in different CVD cohorts [3]. Thus, current CVD clinical guidelines recommend 30 to 60 min of moderate to high-intensity aerobic exercise and resistance training with a frequency of 3–5 sessions per week for patients with CVD [4].

Regarding the recommendations of the experts on the appropriate duration of a cardiovascular rehabilitation program for CVD patients, these are still unclear [5]. For example, a position statement recommends that the minimum number of sessions of an effective cardiovascular rehabilitation program is at least 24, and ideally 36 sessions [6]. However, short-term programs (<24 sessions) have shown similar effectiveness for increasing CRF [7], reducing cardiovascular risk [8], and improving QoL, anxiety and depressive symptoms in CVD [9,10]. The evidence then suggests that the effect of exercise training depends on the number of sessions independently of the volume and session duration with the aims of cardiovascular rehabilitation [8]. Given the high prevalence of CVD worldwide, optimizing the dose–effect relationship in CCR is paramount for improving cardiovascular health in these cohorts.

Although the main objective of CCR is reducing cardiovascular risk [11], none of the recommendations regarding a number of sessions are based on the assessment of the cardiovascular risk at baseline. According to Barbosa et al. (2014) [12], risk stratification is essential for prescribing exercise training to these cohorts. Relevant expert panels such as the American Association of Cardiovascular and Pulmonary Rehabilitation (AACVPR) [13] measure cardiovascular risk. According to parameters, the risk will increase with low CRF, reduced left ventricular ejection fraction, abnormal cardiac symptoms and signs of physical exertion, complex dysrhythmias, hemodynamic alterations, and emotional parameters such as depression. To our knowledge, these factors have the potential to reduce the performance of physical effort and limit the direct benefits of a CCR program. Therefore, knowing the cardiovascular risk level before the start of a CCR program can be useful information for planning the appropriate number of sessions to achieve the health-related goals of a cardiovascular rehabilitation program.

On the other hand, it is well known that the same exercise training regime applied to a particular sample could promote a wide interindividual variability to exercise training [14,15]. This means that some patients could be high-responders (HRs), moderate-responders (MRs), low-responders (LRs) or non-responders (NRs) for improving a particular health outcome. Different methods have been described for NRs classification, including the statistical typical error of measurement [14], clinical cut-off points [16], and quartile categorization of the exercise training results [17]. This variation in exercise training effects has been shown for anthropometric/body composition, cardiometabolic, and physical fitness/cardiorespiratory performance tests previously [15,18]. The main part of these studies has been applied in physically inactive adults, overweight, obese, morbidly obese, prehypertensive, or hypertensive adults and in populations with poor glucose control that represent the wide comorbidities of the CVD patient’s profile. 

However, little is known about the interindividual variability to exercise training in CVD cohorts, and particularly in heart valve surgery (HVS), heart failure with reduced ejection fraction (HFrEF), post-acute myocardial infarction (post-AMI), and in coronary artery disease (CAD) patients. Thus, the purpose of this study was to describe the average effects and the interindividual variability in a CCR program prescribed according to cardiovascular risk stratification on cardiorespiratory fitness (CRF), anthropometric/body composition, quality of life and emotional health in four CVD profiles.

## 2. Materials and Methods

### 2.1. Participants

Between January 2019 and February 2020 an anonymized database was obtained from a Cardiac Rehabilitation Program at the Dr. Jorge Kaplan Foundation. Patients with HVS, HFrEF, post-AMI, and CAD were included. Inclusion criteria were (a) ≥18 years, (b) completion of the concurrent exercise training regime, (c) completion of all baseline and final evaluations of CRF with a cardiopulmonary exercise test (CPET). The study excluded individuals with (a) dementia or encephalic vascular disease diagnoses, (b) hearing loss, (c) a non-maximal CPET (respiratory exchange rate <1.10), and (d) an implanted cardiac assistive device. 

One hundred and forty patients (*n* = 107 men; 76.4%) were included in the study. Thirty-three patients were diagnosed with HVS, (*n* = 22) with HFrEF, (*n* = 40) were post-AMI, and (*n* = 45) with CAD (Table 1). Dates of admission and discharge from rehabilitation, and information such as the number of sessions attended, CRF, anthropometric/body composition, QoL, and the emotional parameters (anxiety and depression) were obtained. Anonymization and data protection procedures were approved by an accredited ethics committee (resolution code:06/2020).

### 2.2. Procedures

On admission, participants attended a nursing session where clinical information was collected and a general physical examination was performed to exclude musculoskeletal or balance limitations. Then, participants were referred to a CPET to assess the CRF. The concurrent exercise training guided by a physical therapist consisted of 12, 24 or 32 sessions (3–4 times per week) for individuals with mild, moderate and sever cardiovascular risk according to the AACVPR [19], respectively. Additionally, all participants received three sessions of individual nutritional counseling and psychological support, including anthropometric/body composition and health related QoL/anxiety/depression measurements, respectively. Additionally, an educational module on healthy lifestyle habits and a workshop for the acquisition of stress control strategies were performed by patients. Upon completion of the program all measurements were repeated at post-test.

### 2.3. Concurrent Exercise Training

A low to moderate intensity warm-up lasting 5 min was performed. Then, 20–40 min of continuous aerobic exercise using the power output (PO) achieved during anaerobic threshold (AT). Training load was increased by 10% after 1 week, according to each patient’s perception of exertion (10–14 Borg’s scale). If there were no abnormal cardiocirculatory or electrocardiographic signs or symptoms, aerobic exercises were performed in interval mode. For the latter, each session included 8 intervals of 3 min (110–120% PO at AT) and 8 intervals of 2 min of active recovery (70–80% PO at AT).

Subsequently, 10–20 min of functional training with free weights or elastic bands for beginners (i.e., red, or green) was performed. At least 7 types of exercises involving upper extremities (e.g., one-arm dumbbell snatch, biceps curl, lateral raise, shoulder press), core muscles (e.g., plank, twist, high-low band chop), and lower extremities (e.g., burpees, crawl, squat, squat jump, hip extension) were performed. Each exercise included 4 sets of 30 s of continuous work, interspersed with pauses of 15 s. The session ended with 5 min of stretching and controlled breathing. At all times, participants were monitored with one-lead electrocardiography, oxygen saturation (SpO_2_), perception of effort and arterial pressures with an integrated cardiovascular rehabilitation system (Ergosana, Schiller, Germany).

### 2.4. Nutritional Counseling 

The patients attended the nutritionist for an evaluation and nutritional counseling. A first session allowed a comprehensive diagnosis to be obtained, based on medical history, body weight and height, waist and hip circumference, body mass index, body composition (fat and muscle mass) by bioimpedance measurement, and the 24 h recall to know the reference diet. For the bioimpedance measurement, patients with cardiac assist devices (e.g., pacemakers) were excluded. Then, nutritional objectives were established together with the patient in order to reduce the caloric intake and improve the protein intake for exercise, among other objectives. They were given a dietary plan aimed at energy adequacy with modification of macronutrient intake according to their own requirements and affinity to food. In the control session (approx. in the middle of the program), progress was monitored. Further counseling or assessment appointments could be arranged if required by the patient. Upon discharge, anthropometry indexes and corporal composition were measured. 

### 2.5. Psychological Support

In the first of three sessions, an admission evaluation on the prevalent symptomatology level (anxious or depressive) and quality of life was carried out. In addition, through an interview, limiting factors (red flags) were detected regarding awareness of risk factors, stress response complications, personality traits, psychosocial problems, and the presence/absence of significant social support. Based on what had been observed, the psychologist advised on the use of strategies for the containment of stressful environmental stimuli and emotional disturbances that may increase cardiovascular risk. A second session aimed at observing progress and/or new possible risk factors on mental health. Upon discharge, symptomatology level and quality of life were measured again. 

### 2.6. Lifestyle Education 

All patients received a one-hour healthy lifestyle education module (by a nurse and dietician) and a one-hour anti-stress theorical–practical workshop (by a psychologist). The education module aimed for patients to integrate knowledge about healthy/unhealthy aspects of food and preparations (e.g., the Mediterranean diet), deleterious effects of tobacco consumption on the organism, and the effects of drugs and their relationship with the benefits on the pathology. An anti-stress workshop included strategies to control the stress symptoms through teaching self-relaxation techniques. 

### 2.7. Measurements

#### 2.7.1. Cardiopulmonary Exercise Test

The exercise test was performed on a cycle-ergometer with electromechanical brake (Ergoselect-100, Schiller, Germany) controlled by software (CS-200, Schiller, Germany). The CPET ended when the subject was unable to maintain the minimum required cadence of 60 rpm, by limiting signs/symptoms such as severe dyspnea, dizziness, pallor, or by the appearance of serious cardiovascular signs such as complex arrhythmias and myocardial ischemia. The gas exchange analyzer (PowerCube, Schiller, Germany) was calibrated for environmental pressure, flow/volume, O_2_, and CO_2_ before each CPET. Peak oxygen uptake (VO_2peak_) was considered as the VO_2_ value obtained at maximum load during CPET and the AT was determined through the v-slope method. Here, CRF outcomes such as relative (VO_2peak_) and VO_2peak_ predicted (VO_2peak-pred_), maximal metabolic equivalent task (MET_max_), absolute (HR_max_) and predicted maximal heart rate (HR_max-pred_ = 220-age), maximal oxygen pulse (ratio between VO_2peak_ and HR_max_), slope of the ventilation minute and carbon dioxide volume ratio (VE·VCO_2slope_^−1^), and maximal power output (PO_max_) were analyzed.

During CPET, electrocardiography, SpO_2_ and blood pressure were recorded. Workload during CPET was preset according to the functional class (FC) of each participant (FCI = 5 W·min^−1^; FCII = 6–7 W·min^−1^; FCIII = 8–9 W·min^−1^; FCIV = 9 W·min^−1^ or more). In some cases, these loads were adjusted to maintain a progressive metabolic adaptation that would allow participants to achieve a test duration close to ~10 min according to previous recommendations [20]. 

#### 2.7.2. Anthropometric and Body Composition Parameters 

Body weight, height, and body mass index (BMI) were obtained using a scale with stadiometer (model-2392, Detecto, Webb City, MO, USA). Waist and hip circumferences, and waist-height ratio, were measured using a metallic tape measure (W6060PM, Lufkin, TX, USA). Total fat mass, visceral fat mass and skeletal muscle mass (SMM) were obtained using a bioimpedance device (InBody270, Biospace Inc., Seoul, Republic of Korea).

#### 2.7.3. Quality of Life, Anxiety, and Depression

The short-form QoL questionnaire (SF-36) contains 8 domains related to (1) physical functioning, (2) role limitations due to physical role, (3) bodily pain, (4) general health perceptions, (5) vitality, (6) social functioning, (7) role limitations due to emotional problems, and (8) emotional role and emotional well-being. The scores on all domains are transformed to a scale from 0 to 100, where the highest score indicates the optimal and the lowest the poorest QoL [21]. Additionally, the Hospital Anxiety and Depression Scale (HADS) was used at baseline and post-CCR [22,23]. The demographic and clinical characteristics of participants in all study groups are presented in Table 1. 

#### 2.7.4. Interindividual Variability to Exercise Training

After the end of the concurrent training intervention, we classified all patients according to those HRs, MRs, LRs or NRs using quartile categorization in each main outcome, as previously [17,24,25]. Thus, we additionally reported each percentage of HRs, MRs, LRs, and NRs, including their delta changes mean in each CVD group, and the mean delta changes (Δ) in each quartile to the anthropometric/body composition (ΔWC, Δbody fat, ΔSMM), and cardiorespiratory fitness outcomes (ΔVO_2peak_, VE·VCO_2slope_^−1^, HR_max-pred_, and PO_max_).

### 2.8. Statistical Analysis

**Figure 1:**
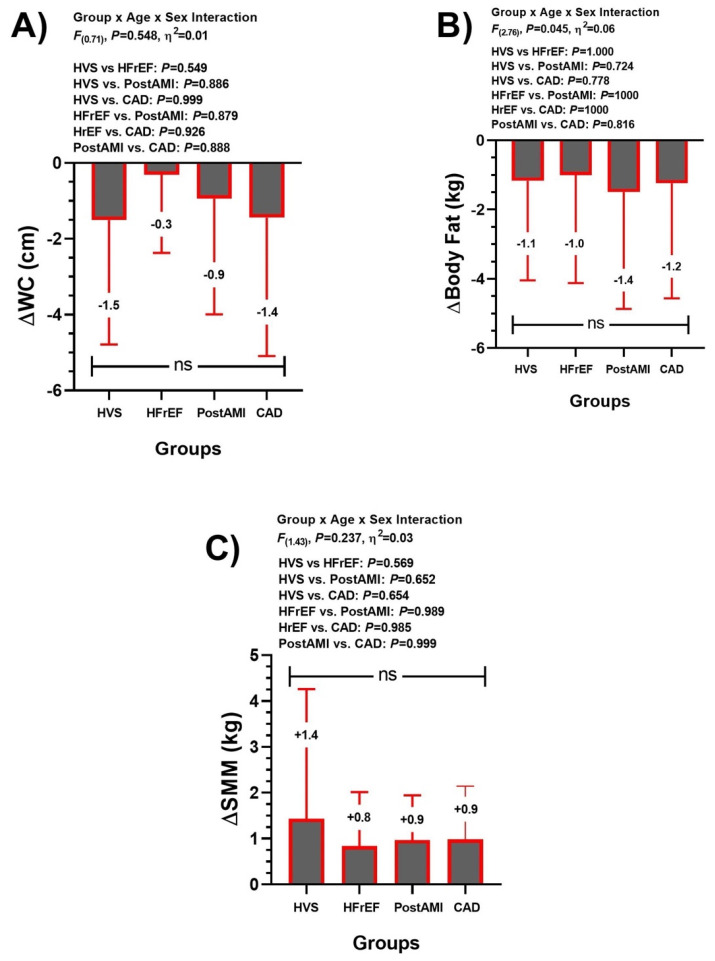
Delta changes (Δ) from pre-post calculation in anthropometric/body composition outcomes (panel (**A**) [WC] waist circumference, panel (**B**) [body fat], and panel (**C**) [SMM] skeletal muscle mass) after a comprehensive outpatient cardiovascular rehabilitation program in heart valve surgery (HVS), heart failure with reduced ejection fraction (HFrEF), post-acute myocardial infarction (post-AMI), and coronary artery disease (CAD) patients. Non-significant (ns) changes among groups.

**Figure 2:**
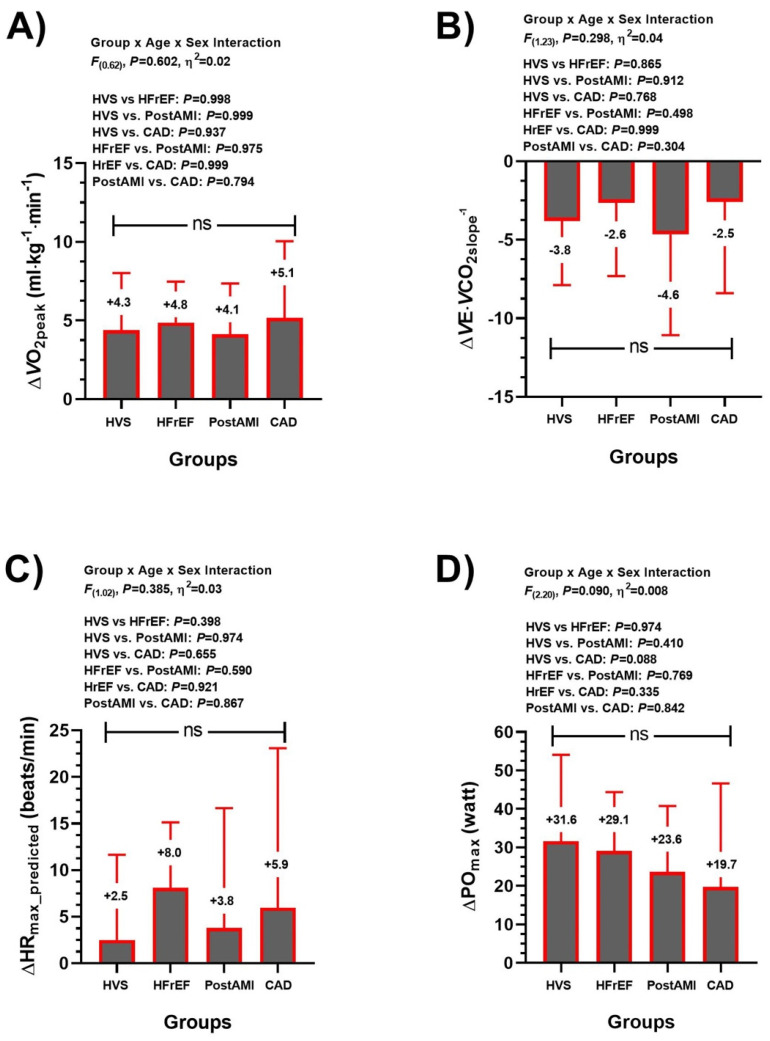
Delta changes (Δ) from pre-post calculation in cardiorespiratory fitness (panel (**A**) [VO_2peak_] maximum oxygen consumption, panel (**B**) [VE·VCO_2slope_^−1^] slope of increase in ventilation in response to CO_2_ production, panel (**C**) [HR_max-pred_] Heart Rate maximum predicted and in performance [PO_max_] Power Output panel (**D**)) parameters. Heart valve surgery (HVS), heart failure with ejection fraction (HFrEF), post-acute myocardial infarction (post-AMI), and in coronary artery disease (CAD) patients. (ns) Non-significant changes among groups.

**Figure 3:**
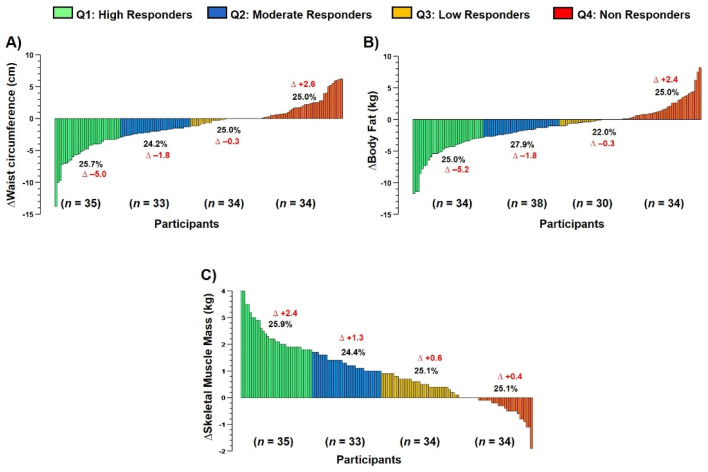
Interindividual variability to anthropometric/body composition outcomes (panel (**A**)) waist circumference, (panel (**B**)) body fat, and (panel (**C**)) skeletal muscle mass after a comprehensive outpatient cardiovascular rehabilitation program based on concurrent exercise training.

**Figure 4:**
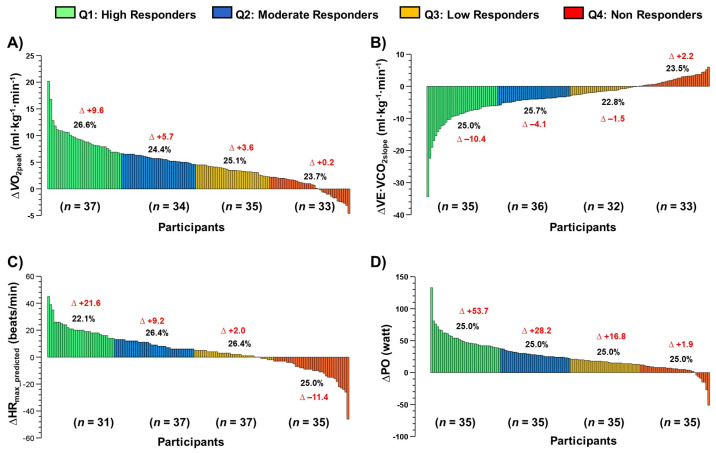
Interindividual variability to cardiorespiratory and performance outcomes (panel (**A**)) peak oxygen consumption [VO_2peak_], (panel (**B**)), slope of the ventilation minute and carbon dioxide volume ratio [VE·VCO_2slope_], (panel (**C**)) heart rate maximum [HR_max_], and (panel (**D**)) maximal power output [PO]. Red values denote ‘average’ delta change by each quartile. Black values denote the percentage of sample of each quartile.

All results are presented as means and standard deviation. A Shapiro–Wilk test was used to test the normality assumption of the variables. We reported the main outcomes anthropometric/body composition (WC, body fat, and SMM), CRF and performance (VO_2peak_, VE·VCO_2slope_^−1^, HR_max_, and PO_max_), QoL and emotional health (anxiety, and depression) in absolute values (Table 2), and calculated delta changes (Δ) to show inter-group interaction (Figure 1 and Figure 2). For baseline testing, a one-way ANOVA was used to compare the differences between groups. For training-induced changes, a repeated measure two-way ANOVA was applied to assess the occurrence of an actual training effect; namely, *p* < 0.05 for the interaction (time×group), as well as to the secondary outcomes. A Sidack’s post hoc test was used for multiple comparisons. Eta partial squared for interaction (Time × Group) was assessed by *η*^2^ obtained from the ANOVA with small (*η*^2^ = 0.01), medium (*η*^2^ = 0.06), and large (*η*^2^ = 0.14) effects defined according to Lakens (2013) [26]. Interindividual variability analysis was carried out using quartile classification only to those main outcomes, and categorizing each sample in HRs, MRs, LRs and NRs (Figure 3 and Figure 4) [17,24]. The prevalence of NRs was described using the comparisons by percentage among each CVD profile group. All statistical analyses were performed with Graph Pad Prism, version 8.0 (GraphPad Software, San Diego, CA, USA) statistical software. The alpha level was set at *p* < 0.05 for statistical significance.

## 3. Results

### 3.1. Anthropometric/Body Composition Group Comparison (Main Outcomes)

Delta changes (Δ) comparisons from pre-post calculations revealed that there were no significant (ns, all *p* > 0.05) differences in ΔWC, ΔBody fat (kg), and ΔSMM among HVS, HFrEF, post-AMI, and CAD groups (Figure 1). 

### 3.2. Cardiorespiratory Performance Group Comparison (Main Outcomes)

Delta changes (Δ) comparisons from pre-post calculations revealed that there were no significant differences (ns, all *p* > 0.05) in ΔVO_2peak_, ΔVE·VCO_2slope_^−1^, ΔHR_max-pred_ and ΔPO_max_ among HVS, HFrEF, post-AMI, and CAD groups (Figure 2).

### 3.3. Training-Induced Changes at Anthropometric/Body Composition (Secondary Outcomes)

At absolute values, there were no significant pre-post changes in body mass in HVF, HFrEF, and CAD except for post-AMI group (∆+1.4 kg, *p* = 0.024), while BMI, visceral fat, and waist-to-height ratio did not show significant changes from baseline (Table 2).

### 3.4. Training-Induced Changes at Cardiorespiratory Fitness (Secondary Outcomes)

After the intervention, METs was significantly increased in HVS (3.5 ± 1.3 to 5.3 ± 1.6), HFrEF (3.8 ± 1.4 to 4.5 ± 1.3), post-AMI (4.2 ± 1.5 to 5.6 ± 1.7), and CAD (3.6 ± 1.1 to 5.0 ± 1.2 mL·kg^−1^·min^−1^), all *p* < 0.001 (Table 2). The O_2_pulse_max_ was significantly increased in HVS (7.6 ± 2.6 to 10.7 ± 3.7, *p* = 0.024), post-AMI (9.3 ± 3.4 to 11.4 ± 3.9, *p* < 0.001), and CAD (8.8 ± 5.3 to 10.7 ± 3.1 mL·beat^−1^, *p* < 0.001) (Table 2). VO_2AT-pred_ (%) was significantly increased in HVS (33.5 ± 7.9 to 47.2 ± 12.7), HFrEF (31.0 ± 10.0 to 35.0 ± 9.3), post-AMI (37.4 ± 10.9 to 46.0 ± 12.2), and CAD (32.8 ± 9.2 to 43.5 ± 13.3 %), all *p* < 0.001 (Table 2). VO_2peak-pred_ was significantly increased in HVS (49.6 ± 12.6 to 74.5 ± 15.1), HFrEF (45.8 ± 15.5 to 56.5 ± 14.6), post-AMI (58.7 ± 16.8 to 74.9 ± 17.7), and CAD (51.1 ± 13.3 to 69.9 ± 16.3 %), all *p* < 0.001 (Table 2).

### 3.5. Training-Induced Changes in Quality of Life and Emotional Health Parameters (Secondary Outcomes)

In delta changes values (Δ), the quality of life parameters had a significant improvement in “Physical functioning” HVS (Δ+17.0), HFrEF (Δ+12.1), post-AMI (Δ+9.8), and CAD (Δ+11.2), “Physical role” HVS (Δ+28.4), HFrEF (Δ+26.8), post-AMI (Δ+25.6), and CAD (Δ+25.3), “Vitality” HVS (Δ+18.4), HFrEF (Δ+14.3), post-AMI (Δ+14.2), and CAD (Δ+10.6), and “Social Functioning” HVS (Δ+20.4), HFrEF (Δ+25.3), post-AMI (Δ+20.4), and CAD (Δ+14.8), with all *p* < 0.001, and without significant interaction among groups (Table 2). In emotional health, all groups had changes with significant improvements in anxiety (HVS Δ−3.6, HFrEF Δ−2.3, post-AMI Δ−3.0, and CAD Δ−3.1) and depression symptoms (HVS Δ−2.8, HFrEF Δ−3.4, post-AMI Δ−3.2, and CAD Δ−2.3) (Table 2).

### 3.6. Interindividual Variability to Anthropometric/Body Composition Parameters (Main Outcomes) 

The interindividual variability to the concurrent exercise training therapy revealed different response to ΔWC, Δbody fat (kg), and ΔSMM (kg) (Figure 3).

### 3.7. Interindividual Variability to Cardiorespiratory Parameters among Groups (Main Outcomes) 

The interindividual variability to the concurrent exercise training therapy revealed different responses to ΔVO_2peak_, VE·VCO_2slope_^−1^, HR_max_ predicted, and PO_max_ (Figure 4).

## 4. Discussion

Considering the aim of this study, which was to describe the average effects and the interindividual variability of a CCR with physical exercise prescribed according to cardiovascular risk stratification on CRF, anthropometric/body composition, QoL and emotional health in four CVD profiles, the present study has four main results: (i) ΔWC, ΔBody fat (kg) are reduced, and ΔSMM (kg) is significantly increased, similarly, without group-differences to the three outcomes (Figure 1); (ii) CRF parameters such as VO_2peak_, VE·VCO_2slope_^−1^, HR_max-pred_, and PO_max_ were significantly improved across groups without group differences (Figure 2); (iii) similar QoL (general health, and mental health [Table 2]), and those emotional parameters (Anxiety and Depression [Table 2]); and finally (iv) despite their improvements post CCR intervention, all these parameters showed a wide interindividual variability (Figure 3 and Figure 4). 

Currently, with aims of cardiovascular rehabilitation, there is no consensus regarding the number of sessions of a CCR program, and the volume time recommended by international experts is widely variable with no consideration about the cardiovascular risk at baseline as our present proposal. Thus, our study summarized that a CCR program that prescribes the number of physical exercise sessions using a cardiovascular risk stratification improves CRF, QoL, and emotional health in individuals with CVD without group differences. This shows that our concurrent training regime applied similar improvements to these parameters in four groups of cardiovascular patients (HVS, HFrEF, post-AMI, and CAD). 

Regarding CRF, a literature review revealed that increases of at least 1.55 METs after CCR in revascularized subjects post-AMI (coronary artery bypass grafting, angioplasty), valve replacement and/or angina were associated with reduced mortality and hospitality re-admission, and it was associated with improvements in QoL parameters [27]. According to these authors, to achieve that work metabolic level, a minimum of 36 exercise sessions over 12 weeks are needed [27]. In our study, similar results on CRF were obtained in CAD, post-AMI and HVS, but not in HFrEF (Table 2). In HFrEF, minor changes were expected, because cardiac dysfunction is the main factor limiting improvements in VO_2peak_. In this sense, increases in CRF between ~0.29 and 0.57 METs (1–2 mL·kg^−1^·min^−1^) positively impact on mortality and hospitalizations in HFrEF [28,29]. In our study, an increase of 0.7 METs (~2.5 mL·kg^−1^·min^−1^) from baseline was observed in HFrEF after 29.8 (4.5) average days of exercise sessions. Recognized cut-off points for cardiovascular dysfunction are recognized in heart failure, such as VO_2peak_ <14.0 mL·kg^−1^·min^−1^ (12.0 mL·kg^−1^·min^−1^ in beta-blocked patients) and VO_2peak-pred_ <50%, which represent a high probability for transplantation/mortality risk in this group [30,31]. Here, 73.9% of HFrEF patients with beta-blocker medication increased the VO_2peak_ and VO_2peak-pred_ to 15.5 mL·kg^−1^·min^−1^ and 56.5%, respectively (data not shown). These changes may be translated into a partial remission in the deterioration of cardiovascular function, and probably a higher survival and lower transplantation probability at 1–2 years [30].

According to the Fick equation, VO_2peak_ depends on the increase in heart rate, stroke volume and arterio-venous oxygen difference (a-vO_2_ diff). The chronotropic response during exertion is the main factor that contributes to the increase in VO_2peak_ [31]. Chronotropic incompetence, i.e., a HR_max_ < 85%, is recognized in heart failure [32], which is considered an independent predictor of adverse cardiovascular events and mortality [33,34]. This effect is caused by beta-adrenergic receptor desensitization induced by chronic sympathetic activation of neurohormonal supercompensation due to decreased cardiac function. In our study, an increase in HR_max-pred_ demonstrated a better chronotropic response during physical exertion in all groups, principally in HFrEF (Figure 2). However, it should be noted that all groups, except HFrEF (HR_max-pred_ 83.1 ± 13.4%), had values above the chronotropic incompetence threshold after CCR (data not shown). Despite this, these results demonstrate a greater cardiac tolerance to effort in all groups, reaching the recommendations for cardiovascular rehabilitation programs [32]. With respect to heart stroke volume, previous studies have shown that O_2_pulse is able to estimate it in healthy subjects and heart failure [35]; however, this could not be recommended for individual use [36]. Here, all groups had significant improvements in O_2_pulse_max_ except in HFrEF. In this sense, unlike the other groups, it is possible that HFrEF achieved greater neurohormonal than structural/functional benefits of cardiac function during exercise after CCR.

Although the evidence shows great complications in post-surgical cardiac function in patients with HVS (low cardiac output syndrome) [37,38], which may limit improvements in VO_2peak_ following a short-term rehabilitation program, significant improvements in O_2_pulse_max_ and VO_2peak_ between HVS, and CAD and post-AMI were detected (Table 2, data partially shown). Similarly, improvements in VO_2peak_ between HVS and coronary bypass after a 3 month cardiovascular rehabilitation program (36 sessions) were previously observed [39]. For the a-vO_2_ diff, it will depend on the relationship to increase blood flow to the active muscle and its ability to extract oxygen. During CPET, an altered a-vO_2_ diff will promote a decrease in VO_2AT-pred_ < 40%, with normal SpO_2_ representing a possible peripheral limitation in the distribution/utilization of oxygen (a-vO_2_ diff reduced) [40]. This reflects a non-ideal oxygen extraction peripheral adaptation, as observed in a study by Dubach et al. (1997) [41], where an exercise program improved cardiac output and arterio-venous difference, but the latter was no greater than the usual care control group. All groups managed to overcome this threshold with significant changes (Table 2), except individuals with HFrEF (with normal maximal SpO_2_). Currently, one of the most promising submaximal indexes obtained from CPET is VE·VCO_2_^−1^_slope_, which represents ventilatory efficiency during submaximal effort, which may be elevated by an increased air dead-space, chemoreceptors numbers, peripheral ergoreceptor activity, and active skeletal muscle mass [42]. Although the HFrEF (32.0 ± 6.7, data not shown) group improved ventilatory efficiency (decreased < 34), this improvement did not reach normal values (25–30) [40]. However, a value lower than 32.9 is associated with a good prognosis in these patients [42]. 

Nutritional counseling and lifestyle education are aimed at controlling body weight, body composition, and cardiometabolic anthropometry indexes (e.g., BMI and waist circumference, among others), in favor of decreasing hyperlipemia, and arterial hypertension. Previous studies have shown moderate or subtle decreases in BMI with nutritional counseling in obese and overweight subjects, respectively [43,44]. However, in our study, there were no significant changes in this variable, but clearly each HVS, HFrEF, and CAD groups showed a trend to increased BMI (Table 2), where we presume that our concurrent training regime promoted fat oxidation (i.e., body fat decreases), but also SMM increases (Figure 1). This is important, as a catabolic state is recognized in these patients, the presence of muscle wasting and cachexia leading to significantly impaired functional capacity [45]. Here, the increases in PO_max_ obtained during CPET after CCR in all groups represents an improvement in muscle work capacity after CCR. Further, a decrease in fat mass (increase in fat-free mass) is related to the control of risk factors such as hypertension, overweight, and dyslipidemia, which have an influence on the development and severity of the CVDs [46]. A recent study observed the effects of nutritional counseling and reported a decrease in BMI of the experimental group in comparison to a control group [47]. In contrast, our results showed a non-significant increase in BMI for HVS, HFrEF and CAD; however, this was related to a greater increase in muscle mass relative to a decrease in fat mass (Figure 1), causing a positive effect on the functional capacity of the individual. In post-AMI, a decrease in body fat contributed to the decrease in BMI; however, the same behavior in the corporal masses was observed (Figure 1). Previously, these changes induced by concurrent exercise have been associated with benefits in CVD [48,49].

The CCR showed benefits in health related QoL components for all diagnoses. This has been previously associated with a decrease in cardiovascular risk, mainly in CAD [50,51].

One aspect to note is the general health item, in which, although there was a significant increase in all groups, these effects did not show at least a minimal improvement, i.e., between 15 and 25 points according to [22]. It is possible that the perception of improvements in general health is acquired once cardiac patients are inserted back into their socio-community activities (e.g., work), as they are finishing a clinical context. Physical functioning relates to the ability to perform strenuous activities, which are restricted in this period for these patients; however, only a minimal improvement was observed in HVS. Results such as physical functioning were found for vitality, which represents daily enthusiasm. Greater enthusiasm with minimal change was only observed in the HVS group, which could be related to the possibility of executing more vigorous activities. In this sense, during CCR it is crucial that the patient discards misconceptions about the safety of physical exercise, which could limit adequate participation in CCR programs. Rather, a permanent anxious state affects the possibility of achieving high levels of physical exertion related to increased physical performance and wellness.

Another relevant aspect was the increased physical role. The great clinical impact observed in all diagnoses (≥25 points according to [22]) is related to the increase in effort tolerance induced by physical training, as a higher score is related to lower problems in the performance of daily activities. Regarding bodily pain, we presume that the continued presence of pain in chronic heart disease, such as HFrEF, is well recognized, and this parameter could not be significantly improved. Comorbidities, lower functional capacity, and depression, among others, favor the presence of pain in these patients [52]. This may explain the minimal clinical change observed in the HFrEF group after CCR. On the other hand, another very relevant factor for the rehabilitation of the cardiovascular patient is the emotional role [53]. It is possible that due to the emotional stress caused by the recent cardiovascular event, and the insecurity due to the life-threatening situation in post-AMI patients, it is possible that they present a high psychological impact [54], which requires a greater number of sessions than those of only 3 months in this study.

Additionally, despite their improvements post CCR in ‘average’ terms, we detected a wide inter-individual variability in the exercise-response in terms of NRs by quartile categorization (Figure 3 and Figure 4). Here, we observed a worsening in several patients in anthropometric/body composition ([ΔWC + 2.6 cm, 25% sample], [ΔBody fat + 2.4 kg, 25% sample], [ΔSMM − 0.4 kg, 25.1% sample]), and cardiorespiratory fitness outcomes ([ΔVO_2max_ + 0.2 mL/kg/min^−1^, 23.7% sample], and [ΔVE·VCO_2slope_^−1^ + 2.2, 25.5% sample], [ΔHR_max-pred_ − 11.4, 25.0% sample_]_, and PO_max_ + 1.9 watt, 25.0% of the sample]). Thus, it is possible to summarize that at least ~25.0% of the patients in each HVS, HFrEF, post-AMI, CAD would not respond to our CCR with concurrent exercise training, and future studies will be developed to detect these potential NRs in early phases of the exercise regime and thus to propose re-orientations of the exercise and others of CCR´s components soon to avoid NRs. In previous studies, there was described a prevalence of NRs not improving WC of 7.2%, body fat 8.6% [55], and SMM of 52.9% [15]. Regarding NRs to CRF outcomes (Figure 2), Bouchard et al. [56] described that, after 6 months of endurance training, the VO_2max_ outcome was only improved in 25% of the sample (*n* = 720), where unfortunately, despite the fast and high trainability of this outcome, a familial compound plays a critical role to their improvement. However, these studies were developed in young sedentary (i.e., physically inactive) participants that are different from our sample of CVD patients, thus acquiring more novelty our findings. 

Finally, our mental health results had clinical improvements in all diagnoses groups that were not minor, which coincides with the improvements in emotional health (anxiety and depression symptoms) observed with the HADS, resulting in normalization of the emotional state (Table 2). For anxiety, the average score in all diagnoses improved from “possible presence of anxiety” to “normal condition” (i.e., questionnaire categorization, data not shown). In depression, all groups decreased their initial values (i.e., questionnaire score, data not shown), despite starting in the “normal range”. Overall, we speculate that the physical health improvements under any CVD condition improve mental parameters in parallel as a consequence of these physical improvements, as has been previously reported after 10 weeks of exercise in CVD patients [57].

It is recognized that cardiovascular surgery (e.g., bypass, valve replacement) favors the detriment of emotional health, due to postoperative pain [54]. However, Sibilitz et al. (2016) [58] showed significant changes in physical fitness, but not in QoL, and anxiety/depression after four months of a CCR program in patients with heart valve surgery. Here, the healthy-lifestyle education intervention was carried out by a professional nurse. In our study, the intervention was performed by a professional psychologist. The latter may have allowed an extra benefit for the patient’s approach strategies. Moreover, in our study, the SF-36 and HADS questionnaires were applied by the psychology professional, unlike Sibilitz et al.’s [58] study, where they developed this section as self-administered. Here, a remission in HVS’s emotional health was observed.

This study is not without limitations. The number of participants with low (4 pers., 2.9%) and moderate (25 pers., 17.9%) cardiovascular risk was lower than those with high risk (111 pers., 79.3%). Another limitation is the possibility of comparing our results with other studies, as previous studies have used treadmill instead of cyclo-ergometer, since it is recognized that a lower VO_2peak_ can be reached in the latter during CPET. Regarding changes in nutritional counseling, there may be some bias because it is well known that lower socioeconomic status is associated with poorer healthy food habit consumption, and we did not evaluate socioeconomic status.

## 5. Conclusions

A CCR program that prescribes the number of exercise sessions using a cardiovascular risk stratification improves CRF, QoL, and psychological health, showing in the average results a wide interindividual variability (~25% of non-responders) in this sample of four CVD profiles of patients. 

## Figures and Tables

**Table 1:** Demographic and clinical characteristics of participants by diagnoses.

Variable	HVS	HFrEF	Post-AMI	CAD
(*n*=)	32	23	40	45
Age, years mean (SD)	60.0 (13.2)	53.0 (14.3)	58.0 (10.6)	63.2 (8.4)
Male, *n* (%)	20 (62.5)	16 (69.6)	31 (77.5)	40 (88.9)
Family history of CVD, *n* (%)	13 (40.6)	12 (52.2)	21 (52.5)	22 (48.9)
Ex-smokers, *n* (%)	26 (81.3)	19 (82.6)	33 (82.5)	37 (82.2)
Ejection fraction % mean (SD)	63.1 (14.6)	28.7 (7.0)	57.2 (12.9)	55.5 (15.6)
Myocardial akinesia, *n* (%)	0 (0.0)	1 (4.4)	4 (10.0)	1 (2.2)
Myocardial hypokinesia, *n* (%)	0 (0.0)	2 (8.7)	1 (2.5)	1 (2.2)
Myocardial dyskinesia, *n* (%)	0 (0.0)	0 (0.0)	0 (0.0)	1 (2.2)
Comorbidities, *n* (%)				
DMNID	3 (9.4)	2 (8.7)	10 (25.0)	10 (22.2)
DMID	4 (12.5)	4 (17.4)	4 (10.0)	6 (13.3)
Dyslipidemia	10 (31.3)	6 (26.1)	32 (57.5)	32 (71.1)
Arterial hypertension	17 (53.1)	11 (47.8)	27 (67.5)	36 (80.0)
Asthma	0 (0.0)	0 (0.0)	1 (2.5)	1 (2.2)
Stroke	1 (3.1)	0 (0.0)	0 (0.0)	1 (2.2)
COPD	0 (0.0)	0 (0.0)	0 (0.0)	1 (2.2)
CKD	0 (0.0)	0 (0.0)	1 (2.5)	2 (4.4)
Medical interventions, *n* (%)				
Arterial bypass	2 (6.3)	1 (4.4)	12 (30.0)	38 (84.4)
PTCA	1 (3.1)	1 (4.4)	25 (62.5)	7 (15.6)
Stent	1 (3.1)	0 (0.0)	13 (32.50)	5 (11.1)
Thrombolysis	0 (0.0)	0 (0.0)	3 (7.5)	1 (2.2)
Pacemaker	3 (9.4)	2 (8.7)	0 (0.0)	1 (2.2)
AICD	0 (0.0)	4 (17.39)	0 (0.0)	0 (0.0)
IRD	0 (0.0)	2 (8.7)	0 (0.0)	0 (0.0)
LVAD	0 (0.0)	1 (4.4)	0 (0.0)	0 (0.0)
Mitral valve surgery	7 (219)	2 (8.7)	1 (2.5)	2 (4.4)
Aorta valve surgery	25 (78.1)	1 (4.4)	1 (2.5)	6 (13.3)
Drugs, *n* (%)				
NSAIDs	19 (59.4)	13 (56.5)	28 (70.0)	32 (71.1)
Analgesic	9 (28.1)	8 (34.8)	16 (40.0)	16 (35.6)
ACE inhibitors	15 (46.9)	11 (47.8)	19 (47.5)	17 (37.8)
ARB	16 (50.0)	9 (39.1)	20 (50.0)	21(46.7)
Betablockers	26 (81.3)	17 (73.9)	28 (70.0)	35 (77.8)
Ca^+2^ blocker receptor	2 (6.5)	1 (4.4)	4 (10.0)	5 (11.1)
Diuretics	8 (25)	22 (96%)	6 (15%)	1 (2%)
Slow K^+^	1 (3.1)	4 (17.4)	0 (0.0)	0 (0.0)
Phosphodiesterase inhibitor	1 (3.1)	1 (4.4)	0 (0.0)	2 (4.4)
Antiarrhythmic	5 (15.6)	7 (30.4)	6 (15.0)	7 (15.6)
Anticoagulants	11 (34.4)	5 (21.7)	14 (35.0)	9 (20.0)
Antiplatelet	6 (18.8)	2 (8.7)	8 (20.0)	12 (26.7)
Antithrombotic	3 (9.4)	2 (8.7)	6 (15.0)	11 (24.4)
Hypoglycemic agents	9 (28.1)	8 (34.8)	9 (22.5)	12 (26.7)
Anti-cholesterol drugs	27 (84.4)	18 (78.3)	33 (82.5)	41 (91.1)

ACE: Angiotensin-converting enzyme, AICD: Automated implantable cardioverter defibrillator, ARB: Angiotensin receptor blocker, EF: Ejection fraction, HVS: Heart valve surgery, HFrEF: Heart failure reduced ejection fraction, post-AMI: after Acute myocardial infarction, CAD: Coronary arterial disease, IRD: Implantable resynchronizer device, LVAD: Left ventricular assist device, NSAIDs: Non-steroidal anti-inflammatory drugs.

**Table 2:** Changes in secondary outcomes of anthropometric, body composition, cardiorespiratory performance, quality of life and emotional role in four different samples of patients with cardiovascular disease participants of a concurrent training exercise program.

	Time	HVS	HFrEF	Post-AMI	CAD	*F*_( )_, *p* Value, *η*^2^
Anthropometric and body composition						
Body mass (kg)	Pre	71.0 ± 15.7	73.2 ± 9.9	76.3 ± 14.3	75.1 ± 14.6	*F*_(0.68)_, *p* = 0.563, 0.01
	Post	71.5 ± 14.6	74.2 ± 10.1	77.7 ± 13.7	75.5 ± 14.3	
	*p*-value	*p* = 0.443	*p* = 0.232	*p* = 0.024	*p =* 0.500	
	∆	+0.5	+1.0	+1.4	+0.4	
BMI (kg·m^−2^)	Pre	26.6 ± 4.7	26.7 ± 4.7	28.2 ± 4.4	27.3 ± 3.2	*F*_(5.99)_, *p* = 0.016, 0.05
	Post	27.3 ± 54.5	27.1 ± 4.5	28.0 ± 4.2	27.5 ± 3.3	
	*p*-value	*p* = 0.245	*p* = 0.482	*p* = 0.068	*p* = 0.137	
	∆	+0.7	+0.4	–0.2	+0.2	
Visceral body fat (%)	Pre	11.0 ± 4.3	9.3 ± 3.9	11.8 ± 4.4	10.3 ± 3.2	*F*_(2.69)_, *p* = 0.104, 0.02
	Post	10.9 ± 4.3	10.7 ± 7.1	10.8 ± 4.1	9.5 ± 3.0	
	*p*-value	*p* = 0.229	*p* = 0.632	*p* = 0.317	*p* = 0.506	
	∆	–0.1	+1.4	–1.0	–0.8	
Waist hip ratio	Pre	0.93 ± 0.0	0.91 ± 0.1	0.95 ± 0.0	0.93 ± 0.0	*F*_(0.59)_, *p* = 0.442, 0.006
	Post	0.93 ± 0.1	0.93 ± 0.1	0.95 ± 0.0	0.93 ± 0.0	
	*p*-value	*p* = 0.597	*p* = 0.830	*p* = 0.716	*p* = 0.073	
	∆	0.0	+0.02	0.0	0.0	
Cardiorespiratory performance						
METs_max_	Pre	3.5 ± 1.3	3.8 ± 1.4	4.2 ± 1.5	3.6 ± 1.1	*F*_(1152)_, *p* < 0.001, 0.52
	Post	5.3 ± 1.6 **	4.5 ± 1.3 **	5.6 ± 1.7 **	5.0 ± 1.2 **	
	*p*-value	*p* < 0.001	*p* < 0.001	*p* < 0.001	*p* < 0.001	
	∆	+1.8	+0.7	+1.4	+1.4	
O_2_pulse_max_ (ml·beat^−1^)	Pre	7.6 ± 2.6	8.1 ± 3.5	9.3 ± 3.4	8.8 ± 5.3	*F*_(5.99)_, *p* = 0.016, 0.05
	Post	10.7 ± 3.7 *	9.3 ± 2.8	11.4 ± 3.9 **	10.7 ± 3.1 **	
	*p*-value	*p* = 0.024	*p* = 0.505	*p* < 0.001	*p* < 0.001	
	∆	+3.1	+1.2	+2.1	+1.9	
VO_2AT-pred_ (%)	Pre	33.5 ± 7.9	31.0 ± 10.0	37.4 ± 10.9	32.8 ± 9.2	*F*_(47.8)_, *p* < 0.001, 0.31
	Post	47.2 ± 12.7 **	35.0 ± 9.3 *	46.0 ± 12.2 *	43.5 ± 13.3 **	
	*p*-value	*p* < 0.001	*p* = 0.037	*p* = 0.004	*p* < 0.001	
	∆	+13.7	+4.0	+8.6	+10.7	
VO_2peak-pred_ (%)	Pre	49.6 ± 12.6	45.8 ± 15.5	58.7 ± 16.8	51.1 ± 13.3	*F*_(122.4)_, *p* < 0.001, 0.53
	Post	74.5 ± 15.1 **	56.5 ± 14.6 **	74.9 ± 17.7 **	69.9 ± 16.3 **	
	*p*-value	*p* < 0.001	*p* < 0.001	*p* < 0.001	*p* < 0.001	
	∆	+24.9	+10.7	+16.2	+18.8	
Quality of life						
General Health	Pre	62.3 ± 17.2	59.3 ± 10.9	63.5 ± 11.0	61.8 ± 11.3	*F*_(038)_, *p* = 0.766, 0.10
	Post	73.5 ± 14.4 **	66.2 ± 12.6	71.3 ± 12.4	68.0 ± 18.3 *	
	*p*-value	*p* < 0.001	*p* < 0.001	*p* < 0.001	*p* < 0.01	
	∆	+11.2	+6.9	+7.8	+6.2	
Mental health	Pre	61.7 ± 16.7	57.6 ± 17.9	64.5 ± 16.1	64.6 ± 18.0	*F*_(076)_, *p* = 0.518, 0.20
	Post	78.1 ± 16.2 **	75.0 ± 14.5 **	84.5 ± 13.1 **	82.9 ± 19.1 **	
	*p*-value	*p* < 0.001	*p* < 0.001	*p* < 0.001	*p* < 0.001	
	∆	+16.4	+17.4	+20.0	+18.3	
Physical functioning	Pre	69.8 ± 15.4	69.8 ± 11.9	75.5 ± 10.4	73.0 ± 12.2	*F*_(214.9)_, *p* = 0606, 0.67
	Post	86.8 ± 7.4 **	81.9 ± 9.7 **	85.3 ± 15.9 **	84.2 ± 16.4 **	
	*p*-value	*p* < 0.001	*p* < 0.001	*p* < 0.001	*p* < 0.001	
	∆	+17.0	+12.1	+9.8	+11.2	
Role physical	Pre	40.5 ± 28.1	36.3 ± 22.8	52.0 ± 25.1	42.0 ± 23	*F*_(151.1)_, *p* = 0.929, 0.59
	Post	68.9 ± 19.0 **	63.1 ± 24.4 **	77.6 ± 18.3 **	67.3 ± 22.9 **	
	*p*-value	*p* < 0.001	*p* < 0.001	*p* < 0.001	*p* < 0.001	
	∆	+28.4	+26.8	+25.6	+25.3	
Bodily pain	Pre	65.0 ± 22.6	76.4 ± 22.4	74.4 ± 192	70.5 ± 16	*F*_(8.92)_, *p* = 0.729, 0.07
	Post	96.3 ± 17.7	96.8 ± 10.8	93.3 ± 14.6	96.2 ± 16.1	
	*p*-value	*p* = 0.066	*p* = 0.071	*p* = 0.063	*p* = 0.814	
	∆	+31.3	+20.4	+18.9	+25.7	
Vitality	Pre	57.5 ± 15.9	53.3 ± 11.8	61.0 ± 15.2	61.2 ± 12.2	*F*_(93.1)_, *p* = 0.892, 0.47
	Post	75.9 ± 15.1 **	67.6 ± 13.4 **	75.2 ± 14.2 **	71.8 ± 18.6 **	
	*p*-value	*p* < 0.001	*p* < 0.001	*p* < 0.001	*p* < 0.001	
	∆	+18.4	+14.3	+14.2	+10.6	
Social functioning	Pre	57.6 ± 24.2	52.1 ± 20.4	61.4 ± 20.4	57.8 ± 22.4	*F*_(86.2)_, *p* = 0.532, 0.45
	Post	78.0 ± 22.1 **	77.4 ± 21.9 **	81.8 ± 20.6 **	72.6 ± 25.5 **	
	*p*-value	*p* < 0.001	*p* < 0.001	*p* < 0.001	*p* < 0.001	
	∆	+20.4	+25.3	+20.4	+14.8	
Emotional health						
Anxiety	Pre	9.9 ± 2.1	9.9 ± 2.5	8.9 ± 2.1	9.1 ± 2.4	*F*_(79.9)_, *p* = 0.657, 0.43
	Post	6.3 ± 3.0 **	7.6 ± 3.4 *	5.9 ± 2.0 **	6.0 ± 2.2 **	
	*p*-value	*p* < 0.001	*p* = 0.003	*p* < 0.001	*p* < 0.001	
	∆	–3.6	–2.3	–3.0	–3.1	
Depression	Pre	6.3 ± 3.0	7.6 ± 3.4	6.7 ± 3.2	5.8 ± 0.9	*F*_(148.)_, *p* = 0.890, 0.58
	Post	3.5 ± 2.2 **	4.2 ± 2.2 **	3.5 ± 2.2 **	3.5 ± 2.2 **	
	*p*-value	*p* < 0.001	*p* < 0.001	*p* < 0.001	*p* < 0.001	
	∆	−2.8	−3.4	−3.2	−2.3	

(BMI) Body mass index, (METs_ma_) Metabolic equivalent task, (O_2_pulse_max_) maximum oxygen pulse, (VO_2AT-pred_) predicted oxygen consumption at the anaerobic threshold, (VO_2peak-pred_) predicted maximum oxygen consumption. (*) Denotes significant pre-post changes at *p* < 0.05. (**) Denotes significant pre-post changes at *p* < 0.01.

## Data Availability

The data that support the findings of this study are available from the corresponding author upon reasonable request.
